# Post-lingual non-syndromic hearing loss phenotype: a polygenic case with 2 biallelic mutations in *MYO15A* and *MITF*

**DOI:** 10.1186/s12881-019-0942-4

**Published:** 2020-01-02

**Authors:** Athar Khalil, Samer Bou Karroum, Rana Barake, Gabriel Dunya, Samer Abou-Rizk, Amina Kamar, Georges Nemer, Marc Bassim

**Affiliations:** 1 Departments of Biochemistry and Molecular Genetics, Faculty of Medicine, American University of Beirut, Beirut, Lebanon; 2 Otolaryngology – Head and Neck Surgery, Faculty of Medicine, American University of Beirut, Beirut, Lebanon; 3 Genomics and Precision Medicine Program, College of Health and Life Siences, Hamad Bin Khalifa University, Doha, Qatar

**Keywords:** Congenital hearing loss, Non-syndromic hearing loss, *MITF*, *MYO15A*, Whole exome sequencing

## Abstract

**Background:**

Hearing loss (HL) represents the most common congenital sensory impairment with an incidence of 1–5 per 1000 live births. Non-syndromic hearing loss (NSHL) is an isolated finding that is not part of any other disorder accounting for 70% of all genetic hearing loss cases.

**Methods:**

In the current study, we reported a polygenic mode of inheritance in an NSHL consanguineous family using exome sequencing technology and we evaluated the possible effect of the detected single nucleotide variants (SNVs) using in silico methods.

**Results:**

Two bi-allelic SNVs were detected in the affected patients; a *MYO15A* (. p.V485A) variant, and a novel *MITF* (p.P338L) variant. Along with these homozygous mutations, we detected two heterozygous variants in well described hearing loss genes (*MYO7A* and *MYH14*). The novel MITF p. Pro338Leu missense mutation was predicted to change the protein structure and function.

**Conclusion:**

A novel *MITF* mutation along with a previously described *MYO15A* mutation segregate with an autosomal recessive non-syndromic HL case with a post-lingual onset. The findings highlight the importance of carrying whole exome sequencing for a comprehensive assessment of HL genetic heterogeneity.

## Background

With a prevalence of 1 to 5 per 1000 births, hearing loss (HL) represents the most common congenital sensory impairment. Congenital hearing loss could be either due to hereditary/non-hereditary genetic factors, or due to certain complications during pregnancy and childbirth [1]. Most of the cases (~ 60%) are attributed to genetic causes with more than 150 genes identified to be associated with either syndromic or non-syndromic form of this disease [2, 3]. Non-syndromic hearing loss (NSHL) accounts for 70% of genetic HL cases that are usually not associated with other signs and symptoms. NSHL can be inherited either in an autosomal recessive manner (75–80%), autosomal dominant manner (20–25%), X- linked or in rare cases by mitochondrial inheritance (1–2%) [4]. Up to date, over 115 genes have been linked to non-syndromic HL with *GJB2, SLC26A4, MYO15A, OTOF*, and *CDH23* being considered as the most commonly identified genes. Some of these genes were shown to be associated with both recessive and dominant form of the disease [5, 6].

With the advent of next-generation sequencing (NGS), genetic mapping within large, clinically well-characterized families with NSHL provides a powerful approach for mapping critical chromosomal intervals which when mutated could be responsible for this phenotype. In the Middle East, the high rate of consanguineous marriages favors the incidence of autosomal recessive diseases such as that of NSHL [7]. Unfortunately, despite this high prevalence, the needed genetic linkage studies using NGS technologies are still not very well established [8].

In this study, we report a polygenic mode of inheritance in an NSHL consanguineous family using exome sequencing analysis. Accordingly, we propose for the first time the involvement of a novel *MITF* variant along with a previously described *MYO15A* mutation in non-syndromic HL disease with post-lingual onset.

## Methods

### Subjects

Two young siblings presented to the Department of Otolaryngology - Head and Neck Surgery at American University of Beirut (AUB) with a complaint of late-onset HL. These patients, along with their consanguineous family, were included in the ongoing study of the genetic basis of HL in Lebanon. Family members received a complete otolaryngologic examination, in addition to pure tone audiometry testing. They were also referred to Ophthalmology, Cardiology and Nephrology for identification of possible other congenital abnormalities and ruling out syndromic HL. A follow-up examination was done for one available affected patient (II.5) and her parents after 4 years from the first visit. The study was approved by the Institutional Review Board (IRB) at the American University of Beirut (protocol number:OTO.MB1.02).

### Exome sequencing

Blood samples were collected from the family members and DNA extraction was performed using the QIAamp Blood Midi Kit (Qiagen Sciences, Inc., Germantown, MD), using the manufacturer’s instructions. DNA quantification was also performed through the NanoDrop (Thermo Fisher Scientific, Inc., Waltham, MA) at the molecular core facility at AUB. One microgram of coded DNA samples from both parents and the two patients were shipped to Macrogen (South Korea), where exome sequencing was performed using the V5 SureSelect Target Enrichment Capture system from Agilent on a HiSeq 4000 platform from Illumina.

### Data analysis

Primary analysis was done at Macrogen. Generated FASTQ files were mapped to the reference genome using the SureCall software from Agilent technologies. The Illimuna Variant Studio was used for annotation and variant calls. The Integrative Genomics Viewer (IGV) was also used as a high-performance visualization tool for genomic annotations [9]. To assess the pathogenicity of possible candidates, we used SIFT (http://sift. jcvi.org/), PolyPhen2 (http://genetics.bwh.harvard.edu/ pph2/),MutationTaster (http://www.mutationtaster.org/), and GERP++ (http://mendel.stanford.edu/ SidowLab/downloads/gerp/) scores to predict deleterious variants. To predict the effect of the detected mutations on the protein structure and stability, we used DUET software (http://biosig.unimelb.edu.au/duet/stability).

## Results

### Clinical manifestation

**Fig. 1:**
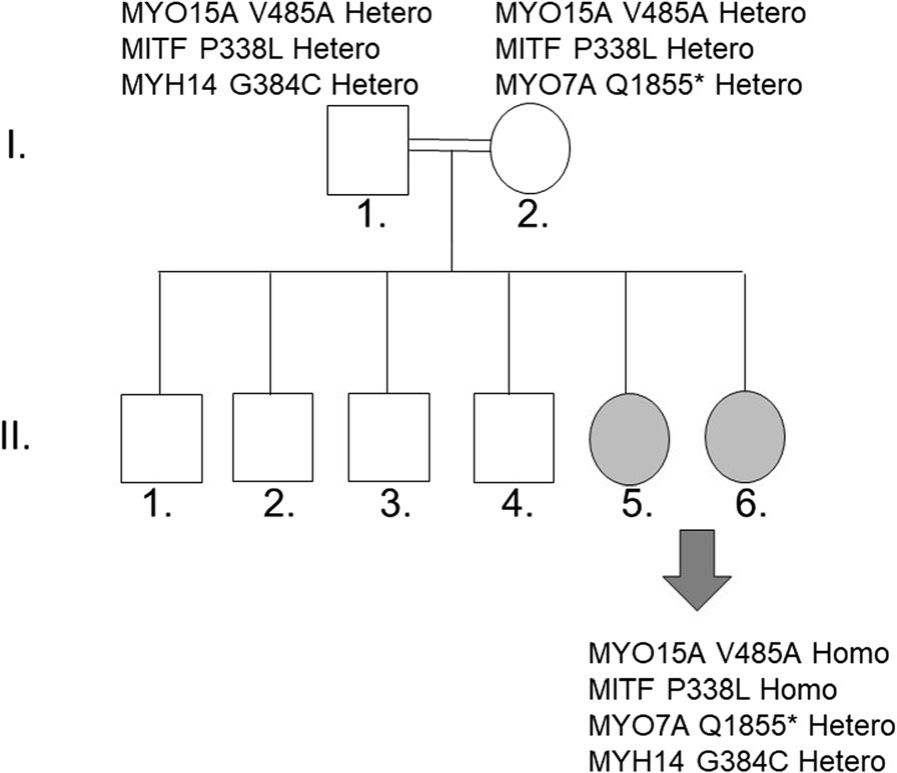
Family’s phenotype and genotype. The pedigree *of* the enrolled family, with affected individuals marked in grey. Possible causative variants of the affected sisters and those of the parents are listed

**Fig. 2:**
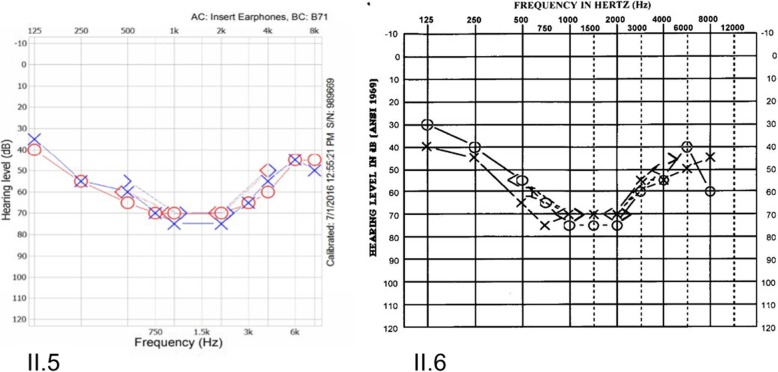
Audiograms of the affected probands. The audiograms show mild to severe progressive hearing loss in both ears for both affected individuals (II.5) and (II.6). The audiograms were taken at the time of diagnosis

The family consists of consanguineous parents with two sisters diagnosed with post-lingual hearing impairment and four unaffected brothers (Fig. 1). HL was noted in the two sisters (II.5/II.6) at the age of six and twelve, respectively. Physical examination did not demonstrate any dysmorphic features suggestive of a syndromic disease. Both patients were reported not to have any pigmentary changes in hair, eyes, or skin. No visual complaints including night blindness, visual field loss and decrease in central vision were detected. Audiogram analysis of this family revealed that the two siblings had a bilateral HL. Puretone audiometry for patients revealed approximately similar pattern of a “cookie-bite audiogram” with mild HL in the low frequencies, sloping to borderline severe in mid frequencies, and rising to moderate in high frequencies (Fig. 2). Word discrimination score was excellent for both patients at the time of referral.

A follow-up audiogram for the patient (II.5) indicated a stable hearing after 4 years from the initial diagnosis. In addition no features of any syndromic disease that are usually initiated after puberty were detected.

### Mutational analysis

Exome sequencing of the four family members achieved approximately (95%) mean exome coverage, at coverage of (8X). From a total number of around 58,000 variants, we only analyzed those that occur in the coding regions of the genes. We filtered variants via a list of 155 genes used for clinical diagnosis of HL while including only missense, frameshift, splice and stop gained alterations with a minor allele frequency (MAF) of < 0.01 (Additional file 1: Table S1). Possible causative variants for each patient were summarized in (Additional file 2: Tables S2 and Additional file 3: Tables S3). The strong candidate variants that might underlie the mild to moderate NSHL in the two patients were those detected in *MITF, MYO15A, MYO7A*, and *MYH14* genes (Fig. 1, 10].

Two bi-allelic single nucleotide variants (SNVs) were detected in the two patients; a previously described *MYO15A* (NM_016239.3:c.1454 T > C) mutation and a novel *MITF* variant (NM_198159.2:c.1013C > T) resulting in the missense mutations p.V485A and p.P338L respectively (Additional file 2: Table S2). Moreover, on the top of the variants that were detected amongst the known HL genes were: 1- a mono-allelic variant in *MYO7A* (NM_000260.3:c.5563C > T) resulting in the nonsense mutation p.Q1855* inherited from the mother, and2- a heterozygous variant in *MYH14* (NM_001145809.1:c.1150G > T) inherited from the father. (Fig. 1 and Additional file 2: Table S2).

Finally, a search for unbiased bi-allelic mutations in the family did not yield additional variants with a MAF < 1% except for *TRPV2* (rs756373391). The latter is a close member of the *TRPV4* gene that is implicated in some cases of HL (Additional file 4**:** Tables S4 and Additional file 5: Tables S5).

### In silico prediction and modulation for the novel *MITF* variant

**Fig. 3:**
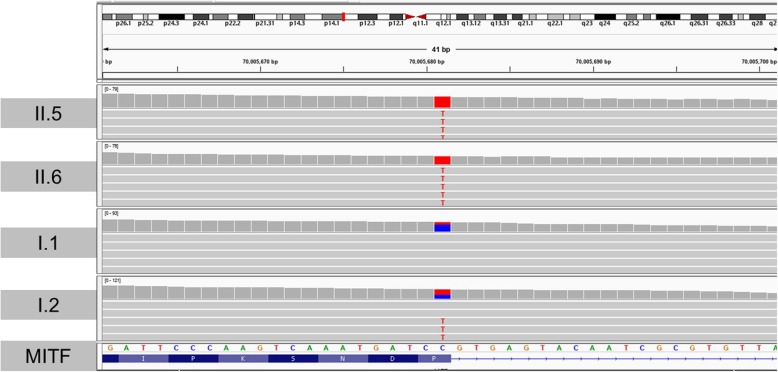
Chromosomal localization of the MITF missense mutation. The NM_198159.2:c.1013C > T variant on chromosome 3 is visualized Using the IGV software. Both parents (I.1 and I.2) carry the heterozygous form (blue and red), whereas both affected daughters carry the homozygous form (red). The amino acids are shown in the lower panel below their corresponding codons, whereas a straight blue line was shown under the nucleotides that correspond to the intronic region

**Fig. 4:**
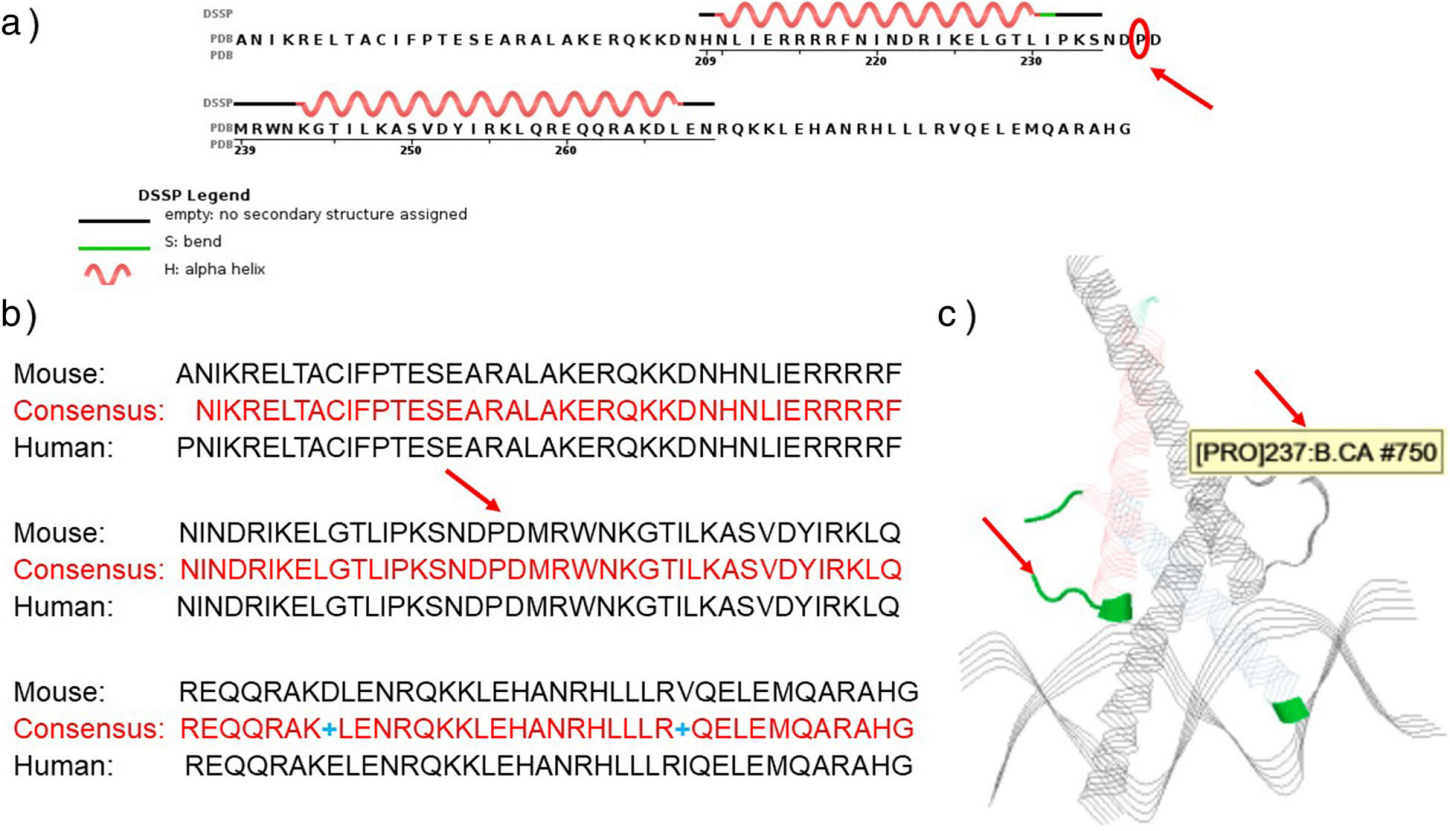
Structural Characterization of the P338 residue. The mouse bHLH amino acid sequence (**a**) used for depicting the crystal structure of MITF bound to DNA showing the position of the corresponding P338 residue (red circle and arrow) is highly identical to the human sequence (**b**). The position of the proline residue at position 338 (referred to as Pro 237) is to the outside of the interface of the dimerization interface between two molecules of the mouse MITF bHLH domain (**c**). (adapted from https://www.rcsb.org/structure/4ATI)

**Fig. 5:**
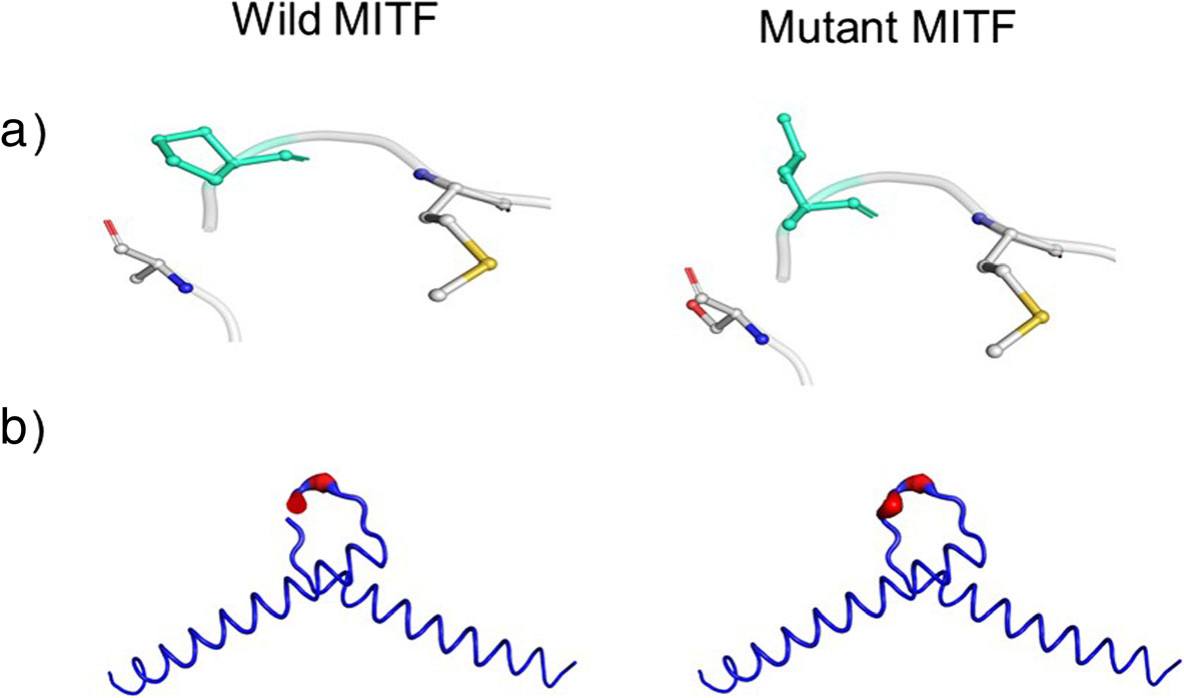
Protein structure prediction of the novel *MITF* variant (p.P338L). In silico modeling (**a**) the effect of the MITF mutation using the DUET software shows a general destabilization of the structure (**b**). Wild-type and mutant residues are colored in light-green and are also represented as sticks alongside with the surrounding residues which are involved on any type of interactions (**a**). The magnitude of the fluctuation is represented by thin to thick tube colored blue (low), white (moderate) and red (high) (**b**)

We focused our analysis on the NM_198159.2:c.1013C > T variant in *MITF* because it lies on the boundary of exon8 and as such could lead either to a missense mutation and/or alternative splicing (Fig. 3). We evaluated the possible effect of the = p. Pro338Leu missense variant on the structure and function of the MITF protein using different in silico predictive software. The proline residue at position 338 lies within the α-helix of the bHLH motif domain (Fig. 4). The amino acid substitution in the MITF protein is predicted to be damaging by Polyphen2 (score 1; range 0–1 with 0 = benign and 1 = probably damaging). SIFT predicts that the substitution is tolerated (score 0.92; a score ≤ 0.05 predicts the change to be damaging and > 0.05 predicts it to be tolerated). However, mutation taster predicts the substitution to be disease causing with a probability of 1 (0–1) (Table 1). In order to better assess this perturbation on the protein structure and its DNA binding activity, we performed an in silico protein stimulation assay, using the modelled-crystal structure of the bHLH domain of MITF (Fig. 4a) bound to DNA (PDB#4ATI). Interestingly, both the murine and human MITF proteins shared high identity in their amino acids bHLH domain including the Proline residue at position 338 which is highly conserved among species (Fig. 4b). Molecular modeling predicts that substitution of proline for leucine can destabilize the protein (NMA Based Predictions ΔΔG ENCoM: 0.207 kcal/mol) (Fig. 5). Therefore, it is expected that this missense mutation changes the structure of the protein, thus, affecting protein function either by disrupting its homotypic/heterotypic dimerization, its DNA binding affinities, or its interaction with partners.

**Table 1:** Pathogenicity scores of detected variants assessed by SIFT, PolyPhen2, Mutation Taster, and GERP++ software

Allelic Variant	Zygosity	Amino acid change	SIFT	Polyphen-2	MutationTaster	GERP++
*MITF* NM_198159.2 c.1013C > T	Homozygous	P338L	Tolerated Score:0.92	Probably damaging Score:1	Disease causing Score:1	Conserved Score:5.06
*MYO15A* NM_016239 c.1454 T > C	Homozygous	V485A	Damaging Score:0	Probably damaging Score:0.9	Disease causing Score:0.9	Conserved Score:5.1
*MYH14* NM_001145809 c.1150G > T	Heterozygous	G384C	Damaging Score: 0	Probably damaging Score:1	Disease causing Score:1	Conserved Score:3.42
*MYO7A* NM_000260.3 c.5835 C > T	Heterozygous	Q1855*	NA	NA	Disease causing Score:1	NA

## Discussion

Although consanguinity can facilitate the discovery of novel genes associated with many diseases, yet it challenges the concept of single causative genetic variant [3]. Interestingly, in this study we revealed a polygenic inheritance of NSHL with the liaison of two independent homozygous alterations in well-known HL genes. To the best of our knowledge, this is the first study to report the implication of a novel *MITF* variant in an NSHL case with an autosomal recessive mode of inheritance and a post-lingual onset.

### *MYO15A* and *MITF* homozygous alterations: the dilemma of predictive tools?

*MYO15A* encodes for XVA myosin protein which plays a vital role in the elongation and development of stereocilia and actin filaments. More than forty *MYO15* mutations have been reported in the motor domain of the protein with generally autosomal recessive HL impairment characterized by a profound phenotype at all frequencies [10]. The detected homozygous *MYO15A* mutation, p.V485A, was previously associated with a HL phenotype in an Iranian family [3]. Mutations in the N-terminal domain are thought to be associated with a milder form of HL since they can affect only one of the two major isoforms of the gene [11]. Although the p.V485A mutation is located within the N-terminal domain, our indexed patients suffer from a mild to severe phenotype. In addition, two healthy individuals from the Gnomad Exome database harbor this variant which argue against a major role for this mutation in the affected individuals. Accordingly, we postulate that other players might be linked, in collaboration or independent of *MYO15A*, to the underlying phenotype.

We therefore considered the second shared bi-allelic novel *MITF* gene mutation p. P338L between the two sisters. *MITF* encodes the melanocyte-specific promoter of microphthalmia-associated bHLH transcription factor. A total of more than forty *MITF* mutations have been verified to be disease-causing in patients with either the Waardenburg’s syndrome type 2)WS2) (OMIM#193510) or the Tietz syndrome (OMIM #103500, 12]. Both syndromes are autosomal dominant and are characterized by overlapping phenotypes that encompass HL and pigmentary abnormalities with variable penetrance. To the best of our knowledge, only 2 homozygous *MITF* cases were detected in WS2 and WS4 [13, 14]. In the present study, the detected homozygous p.P338L missense mutation was neither reported in the dbSNP database, nor in the Gnomad Exome/Genome database. It was also absent from more than 300 Lebanese exomes. The heterozygous frequency of this variant is less than 0.00001 in these databases as it is only present in 3 individuals. Since the detected MITF misssense mutation is localized in the bHLH DNA-binding domain and since the *in-silico* analysis revealed a deleterious effect prediction, we accordingly hypothesize that this mutation is disease causing (Table 1). Thus, structural and functional assays are compulsory to assess the effect of this mutation on the ability of *MITF* to heterodimerize, bind DNA, and/or translocate to the nucleus.

Patients who previously presented with HL as the only phenotypic feature were thought to have NSHL. In consequence, only mutations in genes associated with this type of HL were investigated. On the other hand, some SHL cases require special confirmatory tests since the penetrance of secondary features is either incomplete or age dependent. One example is the Usher syndrome which is presented as an NSHL case early-on in life as the onset of the secondary symptom (retinitis pigmentosa) does not appear until puberty. This might cause a false clinical classification of some patients with SHL who can benefit from the appropriate implementation of visual rehabilitation at early stages [6]. Therefore, it is very critical to categorize genes and variants that are either specific to each type or involved in both forms of HL. Another example is the heterozygous *MITF* (p.R110X) variant that was specifically associated with SHL cases but was recently detected in an NSHL case that presented in the absence of WS2 common features (no pigmentary changes in hair, eyes, or skin) [15]. Originally in-vivo studies on the phenotypic variation seen with the different alleles of the mouse *MITF* gene referred to as *mi* gene suggests that mutations in the human *MITF* gene may also manifest themselves in different ways. This proposed a possibility for detecting phenotypes different from the characteristic WS2 phenotype among patients with *MITF* mutations [16]. Combining these facts with our results, we propose expanding the implications of *MITF* variants from syndromic to non-syndromic HL cases while associating it with an autosomal recessive mode of inheritance.

Additionally, it is widely known that most mutations in autosomal dominant loci cause post-lingual hearing impairment (including *MYO7A* and *MYH14*) while mutations in autosomal recessive HL cases with delayed childhood onset are rare clinical findings [17]. Herein, we are the first to propose *MITF* and *MYO15A* variants as autosomal recessive loci causing stable post-lingual hearing impairment rather than a progressive pre-lingual one.

### Polygenic inheritance

Although most genetic deafness cases result from mutations in a single gene, an emerging number of examples are being documented where recessive mutations at two loci are being involved. For example, the digenic interaction that underlies the cause of deafness in individuals carrying a single mutation at the *GJB2* locus along with a deletion in the functionally related *GJB6* gene [18]. Moreover, a study done by Legar.et al. on twelve patients with *MITF* mutations demonstrated a large range of variability in phenotype among these patients which argue for the possible interaction with modifier loci [19]. Herein, we propose a polygenic form of inheritance mainly through the implication of both *MITF* and *MYO15A* variants coupled with two detected heterozygous variants in *MYO7A* and *MYH14* genes. Different compound heterozygous or homozygous mutations related to *MYO7A* have been reported in a variety of autosomal recessive Usher Syndrome families [20]. However, mutations in *MYH14* gene are associated with autosomal dominant hearing impairment [21]. Thus, we speculate an involvement of the detected *MYH14* and *MYO7A* mutations in the observed phenotype but not as the direct independent cause of HL since the parents presented as healthy carriers. Further functional studies are needed to assess the independent and combined effect of these mutations on the development of HL.

Finally, we could not rule out other genetic/epigenetic modifiers that could be associated with the underlying phenotype, especially that a growing number of studies have showed that copy number variation (CNV) is widely encountered in syndromic and non-syndromic HL cases [22–24]. Such studies would require a case-control study with a substantial number of patients with SHL, NSHL, and controls.

## Conclusion

The present study describes a rare form of hereditary non-syndromic autosomal recessive post-lingual sensorineural HL that is associated with polygenic inheritance mode of bi- and mono- allelic variants. In this study, we unraveled the association of a novel *MITF* variant in NSHL along with a previously described mutation in *MYO15A* associated with a mild form of HL. We highlighted the importance of clinical exome sequencing for a comprehensive addressing of genetic heterogeneity of HL and in detecting novel variants associated with NSHL.

## Supplementary information

**Additional file 1: Table S1.** List of hearing loss genes used for filtering variants

**Additional file 2: Table S2.** Filtering results from whole exome sequencing for patient II.5 using the 150 genes panel from Supplementary Table 1

**Additional file 3: Table S3.** Filtering results from whole exome sequencing for patient II.6 using the 150 genes panel from Supplementary Table 1

**Additional file 4: Table S4.** Filtering results from whole exome sequencing for patient II.5 showing only homozygous mutations with a MAF < 5%

**Additional file 5: Table S5.** Filtering results from whole exome sequencing for patient II.6 showing only homozygous mutations with a MAF < 5%.

## Data Availability

The datasets used and analyzed during the current study are available from the corresponding author upon a reasonable request. Exome sequencing files are available for sharing with any researcher or research team through a direct request process to the corresponding authors. The novel *MITF* mutation was submitted to ClinVar under accession number: SCV001035077.

## Abbreviations

HL: Hearing loss
NGS: Next Generation Sequencing
NSHL: Non-syndromic hearing loss
SNVs: Single Nucleotide Variants
WS: Waardenburg’s Syndrome
